# Household Food Waste Reduction Determinants in Hungary: Towards Understanding Responsibility, Awareness, Norms, and Barriers

**DOI:** 10.3390/foods14050728

**Published:** 2025-02-21

**Authors:** Veronika Keller, Szandra Gombos

**Affiliations:** Kautz Gyula Faculty of Economics, Széchenyi István University, 9026 Győr, Hungary; kellerv@sze.hu

**Keywords:** barriers, behavioral intentions, food waste, personal norms, sustainability

## Abstract

Food waste poses a substantial global challenge, with intricate environmental, economic, and ethical ramifications. This study examines household food waste behaviors, emphasizing the interplay of personal responsibility, awareness of consequences, personal norms, and systemic barriers. Employing a thematic analysis of in-depth interviews with 60 households across Hungary’s Central and Western Transdanubian regions, this research explores factors influencing waste-reduction strategies across the pre-, mid-, and post-consumption phases. The thematic analysis of the interview data yielded key themes, including ascription of responsibility (AR), awareness of consequences (AC), personal norms (PN), demographic characteristics (DC), behavioral intentions (FWBI), and barriers (B). Participants identified barriers to waste reduction, such as impulse buying, poor storage practices, and limited knowledge of food rescue initiatives. Incorporating these barriers as a core variable deepens the understanding of systemic challenges, while stage-specific analysis illuminates the evolution of waste-reduction behaviors. These insights will inform targeted interventions, such as community programs, educational campaigns, and technology-driven solutions, including food rescue apps, to foster sustainable consumption. This study’s integration of behavioral theories with actionable strategies provides valuable guidance for policymakers and stakeholders seeking to reduce household food waste on a global scale.

## 1. Introduction

Food waste represents one of the most significant challenges facing the global community, with an estimated 1.05 billion tons generated annually in the retail, catering, and household sectors [1]. In Europe, Germany is the country that generates the greatest quantity of household food waste, with an estimated 6 million metric tons per year. This is followed by France and the United Kingdom [2]. Hungary generates approximately 908,669 metric tons of food waste annually, which equates to an average of 59.85 kg per capita in 2022 [3]. The composition of food waste varies by region. In Hungary, fruits and vegetables account for 25% of wastage, followed by bakery products with 18% [4]. Additionally, pre-prepared food represents a considerable source of waste, with an average of 10.6 kg per capita per year [5]. In order to mitigate the impact of rising food prices, many European consumers are focusing on reducing household food waste. For example, 61% of Portuguese respondents reported employing this strategy [5]. In Hungary, the proportion of minimum wage expenditure on basic foodstuffs reached 23.9% in January 2024, underscoring the economic impact of food prices and waste [6,7].

The issue of food waste reveals complex consumer attitudes and behaviors that are shaped by a variety of ethical, practical, and social factors. A significant proportion of individuals experience feelings of guilt and moral distress when discarding food, acknowledging its ethical implications and the associated social, economic, and environmental consequences [8,9,10,11]. Despite this awareness, some consumers justify food waste on practical grounds, such as mitigating foodborne illness by discarding items past their package dates or ensuring meals remain fresh [9]. However, a pervasive lack of awareness regarding the environmental implications of food waste persists, even among environmentally conscious consumers [12,13]. This incongruity in consumer behavior is indicative of a need for further examination. While consumers may experience feelings of guilt and be aware of the environmental impacts of waste, they nevertheless discard substantial amounts of food for reasons such as spoilage, over-purchasing, improper storage, and forgetfulness [9,12,14]. The complexity of these issues is further compounded by behavioral constraints, including the short shelf life of fresh food, inadequate planning skills, and time constraints [12,14,15]. Household dynamics have been shown to influence food waste attitudes [16]. Higher-income households, families with young children, and those who frequently dine out are more likely to engage in food waste [14]. Age and income further influence attitudes, with younger individuals and affluent households tending to waste more, while older adults and those with lower incomes exhibit greater caution due to financial concerns [14,17]. Cultural differences significantly shape food waste attitudes, influenced by family structure, cultural background, and local policy frameworks [18].

Social norms and ethical dilemmas have been shown to influence efforts to reduce waste. While societal expectations and the desire to set a positive example can motivate individuals, practical barriers often overshadow these aspirations [11,19]. Moreover, individuals frequently face conflicts between convenience and adherence to ethical and environmental norms [11]. Despite the existence of public support for policy interventions aimed at reducing food waste, such as recycling programs and educational campaigns, this support does not consistently translate into behavioral changes [15,18]. However, practical tools that promote better food management, including meal planning and shopping routines, are seen as effective in curbing waste [8,15].

The Theory of Planned Behavior (TPB), developed by Ajzen, has been extensively tested in the context of food waste attitudes [20]. As a widely used framework, TPB has proven effective in explaining the factors influencing intentions and behaviors toward food waste reduction. Canova et al. (2024) [21] expanded upon the TPB framework by identifying key beliefs related to food waste avoidance and incorporating descriptive and moral norms into the model. Using structural equation modeling, their study demonstrated that attitudes, norms, and perceived behavioral control significantly predict intentions, which subsequently influence behavior. Begho and Fadare (2023) [22] emphasized the role of motivations, such as environmental concerns and emotional factors, alongside proactive management practices in shaping food waste reduction intentions and behaviors. Their findings highlighted the effectiveness of combining motivational and management-focused interventions to promote waste reduction strategies. Addressing climate change, Chen (2023) [23] explored household food waste behaviors, identifying intentions, shopping routines, and situational factors as critical predictors. The study further found that motivations and moral norms strongly influence attitudes toward waste reduction. Masdek et al. (2023) [24] examined sustainable food waste management among urban households in Malaysia through an extended TPB framework. Their analysis, based on data from 520 households in Klang Valley, identified two factors shaping attitudes and five factors influencing intentions to adopt practices such as reusing leftovers, separating waste, and composting. Notably, composting was the least adopted practice despite high levels of positive intention. To address this gap, the study recommended a multifaceted approach that includes educational materials, social media campaigns, awareness initiatives, and incentive programs to encourage greater participation in composting.

A study conducted in Kumasi, Ghana, utilized the Norm Activation Model (NAM) in conjunction with the Theory of Planned Behavior (TPB) to explore fruit and vegetable waste management practices among retailers. The findings indicated that personal norms, shaped by the awareness of consequences and ascription of responsibility, significantly influenced retailers’ intentions to manage waste [25]. Building upon this, research in Jiangsu, China, extended the NAM framework to examine household food waste management intentions. The study highlighted that personal norms, along with awareness of consequences and benefits, emerged as pivotal factors in driving reduction intentions. However, a lack of concern was found to negatively affect these intentions [26]. A parallel study in India underscored the pivotal role of community awareness and personal norms, demonstrating that these factors function as mediating variables in the reduction of food waste [27]. In the context of social dining settings, research investigations into over-ordering behaviors integrated NAM with TPB, elucidating that personal norms, propelled by the awareness of consequences and ascription of responsibility, contributed to unfavorable attitudes toward over-ordering, culminating in a reduction of food waste [26]. Within the restaurant context, the research examined the application of NAM to understand customers’ food waste reduction intentions. The study identified that awareness of environmental impacts, ascribed responsibility, and moral norms were significant predictors of such intentions. Additionally, the study identified self-efficacy as a moderating variable [28]. Research conducted in Australia and Singapore explored the influence of culture on the “good provider” norm within NAM. The study revealed that while the “good provider” norm in Australia suppressed food waste reduction intentions, it did not have the same effect in Singapore [26]. Finally, technological innovation was examined in the context of small and medium-sized enterprises (SMEs). The study revealed that personal norms and self-efficacy significantly influence behavioral intentions for food waste management, with technological advancements amplifying these effects [28]. A subsequent study in Indonesia investigated the role of religiosity, demonstrating that it positively influenced personal norms, which in turn enhanced intentions to reduce food waste [28].

A comprehensive review of the extant literature on food waste has identified several perceived barriers that hinder efforts to reduce it [29]. These barriers can be categorized into various factors, including societal, personal, and behavioral aspects (Table 1). Addressing these barriers necessitates a multifaceted approach, encompassing the following: first, raising awareness; second, improving knowledge; and third, creating supportive logistical frameworks. Furthermore, modifying social norms and economic incentives can facilitate the adoption of more sustainable food waste management practices.

A considerable number of consumers are not fully aware of the significant impacts their food waste has on health, the environment, and the economy. This dearth of awareness frequently hinders the implementation of effective food waste management practices, as evidenced by the studies conducted by Amirudin and Gim (2019) [30] and Jevrić and Ćipranić (2023) [31]. Moreover, the dearth of knowledge concerning food recovery and recycling options further exacerbates this issue. Consumers often have a limited understanding of the post-disposal fate of food waste, as emphasized by Trevenen-Jones et al. (2024) [32] and Burton et al. (2016) [33].

In addition to these socio–psychological factors, behavioral and psychological factors have been shown to play a crucial role in influencing food waste. As demonstrated by Wiedmann et al. (2020) [34], variations in moral attitudes, concerns, and intentions to reduce food waste influence consumers’ behaviors and their propensity to adopt waste-reduction practices. Furthermore, negative perceptions of suboptimal food items, including those with unusual appearances or nearing expiration, lead to their rejection, thereby contributing to unnecessary waste, as noted by Stangherlin and de Barcellos (2018) [35], Aschemann-Witzel et al. (2019) [36], and Tufail et al. (2022) [37].

Logistical and practical challenges further complicate efforts to reduce food waste. The time and effort required to track and manage waste, such as the logistical concerns reported by dining services staff regarding the weighing and monitoring of food waste, present significant barriers, as described by Burton et al. (2016) [33]. In densely populated urban areas, issues such as limited space and hygiene concerns add another layer of complexity, hindering food waste recycling initiatives, as explored by Xiao and Siu (2018) [38].

**Table 1:** Perceived barriers.

Category	Description	Authors
Awareness and knowledge	Lack of understanding of food waste impacts and recycling options	Amirudin and Gim (2019) [30]; Jevrić and Ćipranić (2023) [31]; Trevenen-Jones et al. (2024) [32]; Burton et al., (2016) [33]
Behavioral factors	Variations in moral attitudes, concerns, and intentions	Wiedmann et al., (2020) [34]; Stangherlin and de Barcellos (2018) [35]; Aschemann-Witzel et al. (2019) [36];
Logistical challenges	Practical issues with tracking and managing food waste	Xiao and Siu (2018) [38]; Annunziata et al. (2022) [39]
Economic influences	Perceived cost and effort, prioritization of convenience	Aschemann-Witzel et al. (2019) [36]; Tufail et al. (2022) [37]
Social, subjective, and personal norms	Insufficient motivation due to cultural attitudes and social norms	Trevenen-Jones et al. (2024) [32]; Sucheran and Olanrewaju (2021) [40]

The influence of economic and social factors on food waste behaviors is also a salient factor. The economic implications, including the perceived cost and effort associated with reducing waste, often dissuade consumers from adopting sustainable practices. For instance, the prioritization of convenience over sustainability has been demonstrated to lead to higher levels of food waste, as discussed by Stangherlin and de Barcellos (2018) [35] and Annunziata et al. (2022) [39]. Furthermore, social norms and cultural attitudes towards food waste have been shown to have a significant impact on waste management practices. In some contexts, a lack of motivation to sort and recycle waste arises from the prevailing social norms, as highlighted by Trevenen-Jones et al. (2024) [32] and Sucheran and Olanrewaju (2021) [40].

This study underscores the intricacies of food waste behaviors and the multifaceted factors that influence them. A comprehensive strategy that integrates awareness-raising, behavioral incentives, and practical tools, customized to diverse demographic groups and contextual settings, is imperative for the effective reduction of household food waste. This study’s findings indicate that behaviors related to food waste are influenced by personal norms, environmental awareness, and financial concerns. These factors vary across different demographic groups. This study’s key findings include the role of upbringing, community influences, and media in shaping responsibility toward food waste. The analysis of participant behaviors indicates that environmental and financial concerns are primary motivators, leading to strategies such as planning, improved storage methods, and the repurposing of leftovers. Noteworthy impediments identified in this study include impulse buying, time constraints, and a lack of knowledge concerning food rescue.

## 2. Materials and Methods

### 2.1. Study Location and Sampling

This study employed a quota sampling method in Hungary, focusing on the Western Transdanubian and Central Transdanubian regions (comprising a total of nine counties). The sampling aimed to be representative of age and gender, with quotas established based on the 2022 census data (Table 2).

For the first phase of the qualitative research involving in-depth interviews, a total of 60 participants were targeted and distributed according to the following age categories:

- 18%: 15–29 years, 11 participants (five female, six male).
- 35%: 30–49 years, 21 participants (10 female, 11 male).
- 45%: 50–84 years, 27 participants (14 female, 13 male).
- 2%: 85+ years, 1 participant (one female).

Given that the gender ratio and proportion in the two examined regions is nearly equal (in total, females: 51.45%, males 48.55%), the sample was designed to include men and women in almost equal proportions. The sampling also accounted for the gender ratio within each age group based on census data, ensuring that the distribution of men and women in the sample aligns with the population characteristics for each age category. From each county seat and its surrounding agglomeration, five participants were selected, with an additional five participants from other settlements within each county, evenly split between villages and small towns (~50% each).

The participants were selected based on the following criteria:

1. They were identified according to the defined quotas for age, gender, and place of residence.
2. Participants were required to manage their own independent household, including grocery shopping and cooking.
3. Participants had to be at least 18 years old and have voluntarily applied for participation.

### 2.2. Survey Process and Interview Activity

Recruitment efforts ensured diversity in residence, capturing perspectives from urban, suburban, and rural areas while adhering to the representational quotas set for the regions. All potential research participants who applied by the deadline were compiled and screened to ensure that the sample met the predetermined quotas. Once the final participants were selected, they were notified and offered a choice of neutral locations for the interviews, such as a hospitality venue in their locality, or the option to conduct the interview in their own home. The majority of participants (51 individuals, 85%) chose to host the interview in their home. The interviews were conducted at various times of the day and week, depending on participant availability. Conversations were audio-recorded, and written notes were taken on-site. The duration of the interviews ranged from 45 to 60 min.

Prior research by Smith et al. (2021) [41] underscored the pivotal role of environmental awareness and education in food waste reduction initiatives. The researchers hypothesized that individuals with a vested interest in food waste management would possess a favorable attitude toward reducing food waste, which would, in turn, motivate them to engage in behaviors aimed at waste reduction over an extended period. Jones’ (2022) [42] observations concur with this hypothesis, noting that environmentally conscious individuals tend to engage in behaviors such as meal planning, mindful consumption, and reducing food waste in their homes. The participants in this study were selected in accordance with demographic variables specified in the sampling quotas, ensuring the representativeness of the target regions. Environmental awareness was not a criterion for inclusion, allowing for the sample to encompass individuals with varying levels of environmental consciousness, ranging from highly aware to less aware participants. These characteristics rendered them an ideal subject group for this study.

Prior to and during the research, participants were not provided with information regarding food waste in order to prevent any potential bias in their responses. Any questions that emerged during the course of the interviews were addressed only after the interviews had concluded. These subsequent responses were not recorded and were not included in the official duration of the interviews. This methodology was employed to ensure the integrity and objectivity of the study by minimizing the influence of external information on participants’ perspectives. This research focused on exploring the attitudes and behaviors of participants regarding food waste reduction. They were invited to consider practices such as meal planning, portion control, and the reduction of food waste in their daily lives and barriers (Appendix A). The primary focus was on understanding the attitudes toward food waste reduction and identifying potential barriers or challenges that might affect their behavior. At the conclusion of the study, in-depth interviews were conducted to gain insights into participants’ experiences and attitudes toward food waste reduction. The interviews, conducted in November 2024, lasted between 45 and 60 min. All interviews were recorded, transcribed, and reviewed for accuracy.

### 2.3. Data Analysis

Thematic analysis was employed to identify key themes within the data set [43]. The first and second authors conducted a thorough review of each transcript, identifying relevant segments and developing a preliminary code list. Regular meetings were scheduled to address any discrepancies and to develop a codebook that grouped codes into themes. The removal of redundant themes was conducted in collaboration with the research team [44]. A deductive and semantic approach was used to analyze research questions focused on attitudes toward food waste, barriers to waste reduction, and the perceived impact of reducing food waste on the environment [45]. The six-step qualitative data analysis procedure was followed to ensure the highest level of accuracy and precision. The six-step qualitative data analysis procedure was executed as follows: (1) familiarization, (2) coding, (3) generating themes, (4) reviewing themes, (5) defining themes, and (6) reporting [41]. In this study, the researchers employed structured thematic analysis to identify themes related to attitudes concerning food waste. The researchers initiated the analysis by meticulously examining the data, identifying recurring patterns, and applying codes based on participants’ responses. The codes were then grouped into overarching categories corresponding to key attitudes and behaviors, such as initial awareness, adoption of waste-reduction practices, and sustained efforts to minimize food waste.

To ensure the accuracy and consistency of the coding process, the data were analyzed by multiple coders, and the reliability of their coding was evaluated by discussion of the researchers. Any discrepancies were resolved through discussion, and the coding scheme was refined as needed. The researchers meticulously documented the coding process to ensure transparency and employed reflexivity to acknowledge the influence of their perspectives on the analysis. Additionally, the researchers employed data triangulation and participant feedback (member checking) to enhance the credibility of the study. This methodological approach, involving the checking of themes by the study’s participants, contributed to the rigor of the thematic analysis and increased confidence in the study’s conclusions.

## 3. Results

**Figure 1:**
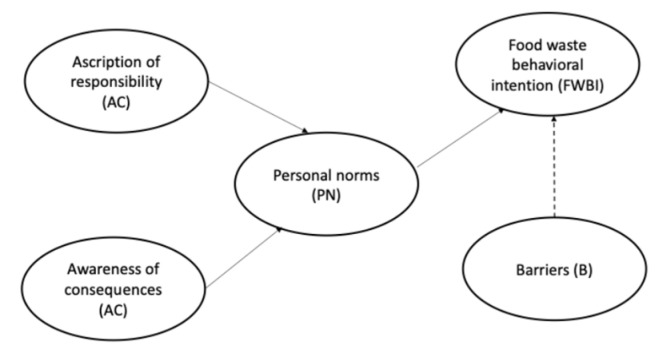
The proposed research model.

The results of this study reveal a complex interplay of factors influencing food waste behaviors, structured around the thematic analysis framework encompassing ascription of responsibility (AR), awareness of consequences (AC), personal norms (PN), behavioral intentions (FWBI), and barriers (B) (Figure 1). These elements collectively elucidate the dynamics of household food waste and the challenges to its minimization.

### 3.1. Ascription of Responsibility (AR)

The analysis of the interview data reveals a multifaceted understanding of responsibility for minimizing food waste (Table 3). The sense of responsibility was not uniform and varied based on contextual considerations. The participants cited environmental concerns, social and moral dimensions—such as awareness of global hunger—and financial pressures like inflation and rising costs as motivators for minimizing waste. However, this sense of responsibility often dissolved in social settings like restaurants, where participants felt detached from the consequences, seeing waste as someone else’s problem.

The participants frequently associated their sense of responsibility with environmental concerns, emphasizing the combined benefits of ecological preservation and financial savings. This alignment between environmental awareness and household economics motivates waste-conscious behavior. Economic constraints further intersect with personal responsibility, encouraging individuals to rethink consumption and disposal habits. Family dynamics and intergenerational education also play a crucial role in shaping food waste practices.

Acknowledging the limits of individual efforts, many participants advocated for collective, community-based solutions, highlighting the importance of shared responsibility. They also pointed to the role of societal structures in sustaining wasteful practices, emphasizing that meaningful change requires a combination of individual, community, and systemic actions.

### 3.2. Awareness of Consequences (AC)

Awareness of the implications of food waste emerged as a crucial determinant of behavior. While some participants admitted to not considering the consequences of waste, others displayed varying degrees of awareness (Table 4). Many had only a superficial understanding, having encountered the concept of food rescue through channels like radio, YouTube, or children’s school programs but lacking in-depth knowledge.

For those with heightened awareness, environmental concerns, such as the impact on future generations and the planet’s livability, were significant motivators. Financial considerations also played a role, as participants recognized the waste of money and resources inherent in discarding food. This awareness directly influenced their subsequent behavioral intentions, underscoring the importance of education and exposure to targeted information.

### 3.3. Personal Norms (PN) and Demographic Characteristics (DC)

Participants frequently identified their upbringing and environment as critical factors shaping their sense of responsibility toward minimizing food waste. Many described being instilled with the ethos of “waste not” during childhood, often influenced by parental behavior and community norms. Additionally, exposure to external influences such as the internet and offline media channels reinforced this mindset. In some cases, children played a reverse role by introducing waste-conscious practices learned through school and community education into their families.

Personal norms, shaped by age, location, financial background, and circumstances, were pivotal in determining attitudes toward food waste. Many participants expressed feelings of guilt or discomfort when discarding food, underscoring the moral weight attached to wastefulness. The perception of what constituted waste also varied, with younger generations and urban residents often having more stringent definitions compared to older or rural participants. These norms created an emotional framework that informed participants’ behavioral intentions, serving as a bridge between their awareness of consequences and their actions.

### 3.4. Food Waste Behavioral Intentions (FWBI)

Participants described a range of pre-, mid-, and post-consumption strategies to minimize food waste, reflecting their internalized norms and awareness (Table 5).

Pre-consumption strategies for reducing food waste can be categorized into four main themes: planning shopping, conscious shopping, conscious storage, and conscious cooking and use. Each of these themes reflects the overarching approaches participants employed to minimize waste, with subthemes highlighting the specific practices within each category. These strategies illustrate the diverse ways households address food waste, shaped by personal attitudes, social norms, and the ascription of responsibility for their actions. The main themes represent the deliberate and structured measures participants adopted to align their consumption habits with waste reduction goals, demonstrating the interplay between intentional behaviors and practical solutions.

*Before and During Consumption Strategies*: Households employed strategies like meticulous purchase planning, including making shopping lists, scheduling purchases, and optimizing quantities to align with consumption patterns. Packaging considerations also played a role, with participants favoring options suited to their needs.

In-store behaviors included adhering to shopping lists, avoiding impulse purchases, and scrutinizing promotions to mitigate psychological pressure. Participants also reported checking expiry dates to ensure optimal utility. Storage practices were another focus area, with participants emphasizing techniques like freezing perishable items, monitoring fridge contents, and rearranging items to prioritize those nearing expiration. Conscious cooking and utilization strategies included not opening more pieces of the same type of product, preparing only necessary quantities, planning menus, optimizing the quantity of prepared food, scheduling the consumption of cooked food, utilizing and maximizing the potential of home-grown ingredients cultivated in personal gardens, and preserving food through a combination of traditional and modern techniques.

*After Consumption*: The distinction between pre-, mid-, and post-consumption strategies is essential in understanding the comprehensive efforts to reduce food waste. While pre-consumption strategies are predominantly preventive in nature, focusing on minimizing waste generation through planning and preparation, post-consumption strategies address the management of leftover food and thus represent a more reactive approach to problem-solving. Within the realm of post-consumption strategies, four main themes can be identified: the consumption of leftovers, the use of leftovers for animals, food donation, and composting. Each of these themes includes specific subthemes, reflecting the diverse approaches participants adopted to manage surplus food and mitigate waste after consumption. These strategies highlight the critical importance of reactive measures in complementing preventive efforts to achieve more sustainable food management practices.

For repurposing leftovers, participants cited examples like converting dry bread into breadcrumbs or transforming ready meals into new dishes. Post-meal practices included freezing leftovers, alternating consumption of refrigerated items, and occasionally consuming food past its expiry date if deemed safe. Additional waste-reduction behaviors extended beyond the kitchen. Participants mentioned donating excess food to neighbors, friends, and food banks, as well as using scraps to feed pets or farm animals. Composting was also practiced, albeit less frequently due to perceived challenges.

### 3.5. Barriers to Food Waste Reduction

Despite the adoption of these strategies, the effectiveness of food waste-reduction practices is often hindered by various barriers that span the pre-, mid-, and post-consumption phases. These obstacles, which align with categories identified in the literature, include awareness and knowledge gaps, behavioral and economic influences, normative pressures, and logistical challenges. Each of these barriers can manifest at different stages of food consumption, reflecting the complex interplay of individual, societal, and systemic factors that impede waste-reducing efforts. Table 6 highlights and summarizes these challenges, exploring how impulsive purchasing, poor storage practices, and post-consumption limitations contribute to food waste. By identifying and categorizing these barriers, the analysis underscores the multifaceted nature of food waste reduction and highlights areas where targeted interventions could enhance the effectiveness of existing strategies.

Before consumption, impulse buying, often driven by promotions and bulk discounts, was a recurring issue. Poor planning or lack of time frequently led to over-purchasing, particularly during festive seasons or in response to external pressures like the COVID-19 pandemic. Stockpiling behaviors, though initially perceived as prudent, often resulted in waste. Inadequate storage practices emerged as a common obstacle in the pre- and post-consumption phases. Participants highlighted issues like forgetting items in the fridge, leading to spoilage. Dairy products, bakery items, and ready meals were particularly vulnerable due to their short shelf lives.

As main post-consumption barriers, participants cited a lack of time and information as critical barriers to implementing waste-reducing practices. A variety of business solutions have been developed to address the behavioral barriers identified. One such solution is the Munch application, which provides a practical, technology-driven solution to these issues [46]. In the future, digital innovations will be more effective and play a greater role in people’s everyday lives [47,48]. Many were unaware of food rescue apps, donation platforms, or proper composting techniques. Additionally, the perceived effort and unappealing nature of composting further deterred adoption.

### 3.6. Practical Implications

This study underscores the interdependence of ascription of responsibility, awareness of consequences, and personal norms in shaping food waste behavioral intentions. Participants with a strong sense of responsibility and heightened awareness were more likely to adopt sustainable practices, influenced by their personal norms. However, barriers like inadequate planning, storage challenges, and informational gaps often curtailed these intentions.

To address these barriers, targeted interventions are necessary. Educational campaigns should focus on enhancing awareness and providing practical guidance on waste reduction. Technological solutions like food rescue apps can bridge informational gaps, while community initiatives can foster a culture of shared responsibility.

In conclusion, reducing household food waste requires a multifaceted approach that integrates individual, social, and structural factors. By addressing barriers and reinforcing motivators, policymakers and stakeholders can create an enabling environment for sustainable food management practices.

### 3.7. Contribution to Theory

**Figure 2:**
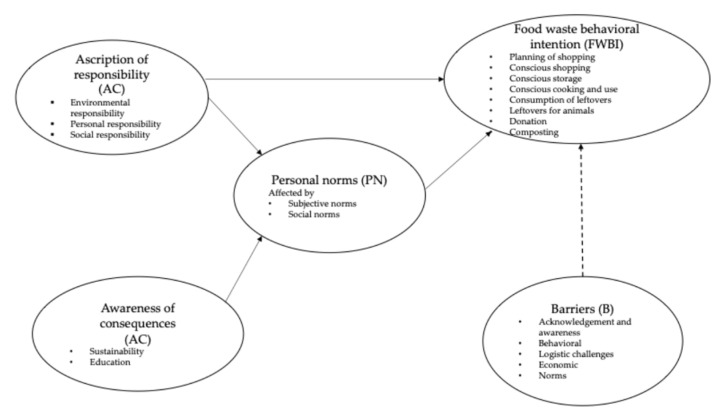
The research model.

The findings of this study contribute to the theoretical understanding of food waste behavioral intentions by proposing a novel model derived from the results of in-depth interviews. This model (Figure 2) highlights how the awareness of consequences and ascription of responsibility influence food waste behavioral intentions through the activation of personal norms, which are further shaped by subjective norms, social norms, and demographic characteristics. The model also establishes a direct link between these influencing factors and the strategies and practices employed to reduce food waste. However, the implementation of these strategies is often impeded by various barriers encountered at different stages of the food waste-reduction process.

The added value of this model lies in its integrative and dynamic nature. It not only combines elements of the Norm Activation Model (NAM) and the Theory of Planned Behavior (TPB) but also incorporates the distinct stages of food waste reduction—pre-, mid-, and post-consumption—providing a more granular understanding of the process. Furthermore, the inclusion of barriers as a new variable enriches the model, addressing a critical gap in previous frameworks. By reflecting the complexity of food waste behaviors and the challenges faced during waste reduction efforts, this model offers a comprehensive theoretical framework that can inform both academic research and practical interventions.

## 4. Discussion

This study contributes to the growing body of literature on household food waste by examining participants’ attitudes and behaviors toward waste reduction. Consistent with previous research, the findings underscore the multifaceted nature of food waste practices, shaped by environmental awareness, financial concerns, and personal norms. This study underscores the significance of addressing both individual and systemic barriers to waste reduction for achieving substantial change.

A notable finding is the role of ascription of responsibility (AR) in motivating waste reduction, aligning with the findings of Stancu et al. (2016) [49], who demonstrated that perceived responsibility strongly influences food waste behaviors. Participants identified motivators such as environmental concerns, economic pressures, and moral considerations (Table 3). However, the study goes beyond these findings by identifying disparities in responsibility attribution in specific contexts, such as social dining, where detachment from consequences often limits accountability.

The role of awareness of consequences (AC) emerged as another critical factor. While prior research, including Alsawah et al. (2022) [8], has linked environmental awareness to waste reduction, this study reveals gaps in participants’ understanding, often shaped by limited media exposure and education. While many participants acknowledged the environmental costs of food waste, their understanding of its social and economic implications was limited (Table 4). This underscores the necessity for targeted educational initiatives that extend beyond superficial awareness, as recommended by Schrank et al. (2023) [50]. Public education campaigns should prioritize enhancing awareness and cultivating personal responsibility, tailoring their approach to specific demographic groups [50,51].

Personal norms (PN) were found to have a significant impact on attitudes and intentions among the study’s participants, aligning with the findings of Stancu et al. (2016) [49] and Begho and Fadare (2023) [22]. Guilt and a sense of moral responsibility were commonly reported, highlighting the emotional aspects of waste reduction. However, this study goes beyond previous research by examining demographic variations and identifying stricter norms among younger participants and urban residents. These findings suggest the potential for targeted, demographic-specific interventions. The participants employed various behavioral intentions (FWBI) and strategies to minimize food waste, including careful shopping, optimized storage, and repurposing leftovers (Table 5). These findings align with previous studies, such as Stefan et al. (2013) [15] and Tóth et al. (2021) [16], which emphasize planning and conscious consumption. However, this study categorizes strategies into pre-, mid-, and post-consumption phases, providing a granular view of practices. Notably, post-consumption strategies such as donating excess food or composting were less prevalent, often hindered by logistical challenges and limited awareness.

This study also identified barriers (B) to waste reduction, consistent with the findings of Zhao and Begho (2024) [29]. Key obstacles included insufficient information about food rescue initiatives, time constraints, and inadequate storage practices (Table 6). Impulse buying and promotional offers have also been identified as contributing factors to over-purchasing, as previously reported by Tufail et al. (2022) [37]. Addressing these barriers through practical solutions, such as user-friendly food rescue apps, streamlined donation processes, and guidance on storage, is essential for fostering sustainable practices. This study makes significant theoretical contributions by integrating elements of the Norm Activation Model (NAM) and the Theory of Planned Behavior (TPB) into a dynamic model that considers the distinct stages of food waste reduction. By incorporating barriers as a central variable, it addresses a critical gap in prior research, which has primarily focused on other aspects of the over-purchasing process.

This study’s three-phase approach—pre-, mid-, and post-consumption—offers a nuanced perspective on the evolution of waste behaviors and barriers. Pre-consumption strategies, such as meal planning, align with the findings of Alsawah et al. (2022) [8], while insights on impulse buying and bulk promotions introduce novel dimensions. During consumption, practices such as portion control resonate with Begho and Fadare (2023) [22], and post-consumption challenges reinforce the need for infrastructure development, particularly in composting and food donation.

## 5. Conclusions

This study provides a comprehensive exploration of the factors influencing household food waste behaviors, emphasizing the roles of personal responsibility, awareness of consequences, personal norms, and systemic barriers. By employing a thematic analysis of in-depth interviews, this research identifies a complex interplay between individual and collective actions and the structural challenges that impede waste reduction efforts. The findings contribute to the existing literature by highlighting the importance of integrating behavioral theories, such as the Norm Activation Model and the Theory of Planned Behavior, with actionable strategies that address stage-specific challenges across the food waste lifecycle. Notably, the inclusion of barriers as a core variable and the detailed examination of pre-, mid-, and post-consumption strategies represent significant theoretical advancements. These insights underline the necessity of tailored interventions, such as targeted education campaigns, community-driven initiatives, and technological solutions like food rescue applications, to bridge the gap between awareness and action. This study’s findings also have practical implications for policymakers and stakeholders, suggesting that regional and demographic differences must be considered to design effective, culturally relevant strategies to mitigate food waste. Future research should build on these findings by incorporating larger, more diverse samples and utilizing mixed-method approaches to validate and expand the theoretical framework presented here (Figure 2).

Household food waste behaviors are significantly influenced by a sense of personal responsibility, which is shaped by factors such as family upbringing, societal norms, and moral frameworks. Participants frequently referenced the influence of their parents, communities, and external media in instilling values of reducing waste. However, a sense of detachment from responsibility emerged in specific contexts, such as dining out or in business environments, where participants perceived the issue to be beyond their individual control. An awareness of the environmental, economic, and ethical ramifications of food waste was a substantial catalyst for behavioral modification. Individuals who exhibited heightened awareness were more inclined to engage in practices aimed at reducing waste, such as conscientious shopping and repurposing leftovers. However, the findings also highlighted a need for more in-depth educational interventions, as the participants’ knowledge about food rescue and waste reduction strategies was limited. This study revealed that personal norms concerning food waste were significantly influenced by age, socio–economic status, and geographical location. Participants in the younger and urban categories exhibited a propensity for more stringent definitions of waste, while those in rural areas frequently demonstrated a greater reliance on pragmatic, hands-on strategies, such as the utilization of food scraps for animal consumption. This study further revealed that personal guilt or discomfort related to food waste was a significant motivator for change, underscoring the moral and emotional weight individuals ascribe to the issue.

Household strategies to minimize food waste included careful meal planning, adherence to shopping lists, and the optimization of storage practices. Furthermore, participants employed various post-consumption strategies, including the freezing of leftovers, the repurposing of food, and the donation of excess food to neighbors or food banks. The behavioral intentions exhibited a direct correlation with the participants’ personal norms and awareness, signifying the potential for interventions aimed at fortifying these links.

Despite the adoption of waste-reducing behaviors, several barriers emerged that hindered participants’ efforts. These included logistical challenges such as time constraints, inadequate storage, and a lack of established routines. Furthermore, impulse buying and promotional offers frequently led to over-purchasing, while a lack of information about food waste reduction tools (e.g., food rescue apps, donation platforms, composting) deterred the broader adoption of sustainable practices.

This present study offers several novel insights into the realm of food waste research. The variation in food waste behaviors based on geographical location (e.g., rural vs. urban) and lifestyle (e.g., farming backgrounds vs. modern consumerism) is a noteworthy finding. Rural participants, particularly those with access to land, exhibited a greater propensity to engage in waste-reduction behaviors, such as feeding food scraps to animals or composting. Conversely, urban participants exhibited a propensity to prioritize mindful shopping and meticulous meal planning. This study underscores a generational divide in attitudes concerning food waste, with younger participants demonstrating heightened awareness and more stringent norms regarding waste. This suggests that educational initiatives targeting younger generations may have a significant impact in the long term, as these habits are more likely to persist into adulthood.

This study noted that participants expressed strong feelings of guilt or discomfort when wasting food, particularly when the food was still considered consumable. This emotional aspect could serve as a unique entry point for interventions, focusing on the moral and ethical dimensions of food waste rather than solely on the environmental or economic consequences. This study revealed that despite participants’ good intentions, barriers such as time constraints, lack of routines, and insufficient information about food donation or composting apps significantly hinder the adoption of sustainable practices. This underscores the necessity for interventions that streamline these practices or facilitate access to resources and information.

This study’s findings can inform the development of policies and public campaigns aimed at reducing food waste. Targeted interventions could focus on providing more accessible and widespread information about food rescue initiatives, donation programs, and composting. Additionally, rural–urban differences and generational attitudes toward food waste could guide region-specific or age-specific initiatives. The role of childhood upbringing in shaping food waste norms is significant; therefore, educational programs that promote sustainability and waste reduction in schools could play a key role in shaping future behaviors. These programs could promote food waste awareness through the curriculum, integrate practical strategies into school lunch programs, and involve students in community-based initiatives, fostering a generation more mindful of food waste. Food retailers and restaurants could leverage the findings to reduce waste at the point of purchase or consumption. The provision of clearer information about food expiration dates, the promotion of smaller portion sizes, or the offering of incentives for sustainable purchasing decisions could assist customers in making more informed choices. Restaurants and food outlets could also adopt waste-reduction practices, such as donation programs or improved waste sorting. The use of technology to address food waste intentions is poised to influence population behavior. Munch offers a compelling illustration of how technological advancements can empower individuals and businesses to make environmentally conscious decisions while concurrently addressing the economic and social dimensions of food waste, including considerations such as affordability and community support. This integration of behavioral insights and digital innovation underscores the potential for scalable solutions in addressing global food waste challenges.

In a nutshell, the individuals and families in the study stand to benefit from heightened awareness and access to tools and resources that facilitate food waste reduction. Practical interventions targeting meal planning, storage practices, and the promotion of leftover utilization could assist households in achieving substantial reductions in food waste.

### Limitations and Future Extension of the Study

Furthermore, this study examined household behaviors but did not address broader systemic factors, such as policies, food distribution, or the role of retailers. The identification of barriers to reducing food waste is an important contribution of the study; however, it lacks a detailed exploration of how to overcome these challenges, especially through digital tools. Finally, this study’s cross-sectional design captures data at one point in time, whereas longitudinal studies could provide more insights into the evolution of food waste behaviors and the impact of interventions. A potential limitation of this study is its reliance on self-reported data, which may be influenced by social desirability bias. Participants may have overreported positive behaviors or underreported wasteful practices to align with perceived social norms. This underscores the necessity for future research to employ objective methods, such as waste audits, to corroborate the findings derived from self-reported data.

Finally, future research should address the limitations of this study, including its small sample size and reliance on self-reported data, which may introduce social desirability bias. To enhance the robustness and applicability of the findings, it is recommended that the study be expanded to include larger sample size and quantitative methods, such as Partial Least Squares Structural Equation Modeling (PLS-SEM), in conjunction with objective waste audits (Figure 2). These steps will support the development of more effective and scalable strategies to reduce food waste. Subsequent research endeavors may entail the exploration of the longitudinal impact of targeted interventions on food waste reduction behaviors, with the objective of assessing their long-term effectiveness. Moreover, extending the study to encompass a more heterogeneous sample from diverse regions and cultural contexts could facilitate a more comprehensive understanding of the factors that influence food waste. The role of emerging technologies, such as smart kitchen appliances and food tracking applications, in supporting food waste reduction efforts could offer valuable insights. Finally, examining the interplay between policy measures and individual behaviors could facilitate the design of more comprehensive strategies to promote sustainable food consumption practices.

## Figures and Tables

**Table 2:** Population age by county based on 2022 census data.

	Central Transdanubian Region (Person)	Western Transdanubian Region (Person)	Central Transdanubian Region (Percentage)	Western Transdanubian Region (Percentage)
**Total**	903,340	841,643	100%	100%
**15–29 years old**	162,826	148,544	18.02%	17.65%
**30–49 years old**	311,892	290,094	34.53%	34.47%
**50–84 years old**	409,418	384,735	45.32%	45.71%
**85+ years old**	19,204	18,270	2.13%	2.17%

**Table 3:** Ascription of responsibility (AR).

Quotes from the Interviews	Subtheme	Theme
*“Wasting less food reduces our environmental impact and saves money.”*	Environmental protection	Environmental responsibility
*“So many people in the world live in hunger, so we don’t throw edible food in the trash.”*	Malnutrition	
*“As an entrepreneur, I know the resources and costs behind a product.”*	Waste of resources	
*“Everything costs more and more, and we can’t afford to waste anymore.”*	Own financial well-being	Personal responsibility
*“For my children’s sake, it’s also important to set an example to avoid waste.”*	Setting an example	
*“Reducing food waste is very important, I personally feel that everyone can do something about it.”*	Individual responsibility	
*“Only together, with community responsibility, can anything be done, I am not enough alone.”*	Shifting responsibility to a larger community level	Social responsibility
*“Obviously this is totally against the consumer society and the capitalist economic system, but I would promote the old ways, reformed and modernized of course.”*	Shifting responsibility to society	

**Table 4:** Awareness of consequences (AC).

Quotes from the Interviews	Subtheme	Theme
*“We are polluting the environment with a lot of unnecessary food purchases, as a large part of the Earth is covered by the huge amount of rubbish we produce.”*	Environmental effect	Sustainability
*“It is very important to maintain at least the current level of the planet’s ability to sustain itself.”*	Sustainability of food supply	
*“The issue of food waste is important, because food supply problems are expected in the future anyway.”*	Malnutrition	
*“In the production of food, we waste a lot of resources like water and energy.”*	Waste of resources	
*“It is important that children learn at an early age, both at home and at school, the value of quality food, so that they can adopt this lifestyle in the future.”*	Future generation	Education
*“I don’t think about food waste because I don’t waste.”*	Ignorance of consequences	

**Table 5:** Food waste behavioral intentions (FWBI).

Before and During Consumption		
Quotes from the Interviews	Subtheme	Theme
*“I’m trying to plan more consciously, to calculate quantities in advance.”*	Planning of optimal quantity	Planning of shopping
*“I always write a shopping list and only buy what’s on it, I don’t deviate from it.”*	Follow shopping list	Conscious shopping
*“When I go to the shop after work on weekdays, I have to concentrate hard not to buy new things that I won’t eat anyway.”*	Avoid impulse buying	
*“We check the contents of the fridge every week or when we buy new things.”*	Continuous review	Conscious storage
*“I try to plan my weekly menus in advance.”*	Menu planning	Conscious cooking and use
*“I try to cook for the three of us so that we all eat something we like, so we don’t have to cook more than one meal and we have food for several days.”*	Optimal cooking quantity and type	
**After Consumption**		
*“I freeze a lot of things and we eat them later.”*	Consumption of leftovers after preservation	Consumption of leftovers
*“If we have leftover soup one day, we ’slide’ it, the next day we eat the soup we cooked, the third day we eat the leftover soup again.”*	Rescheduling the consumption of leftovers	
*“The dogs eat what we don’t use up, so we spend less on their feed.”*	Pets	Leftovers for animals
*“If there is any food left over, we always distribute it among the neighbours.”*	People in physical proximity (neighbors)	Donation
*“There is a Facebook group, I put it up there, if I cook more of something, it usually gets picked up quickly.”*	Online donation	
*“I put vegetable scraps in the composter, coffee grounds on the flowers and banana peels in the flower pots as a source of nutrients.”*	Composting	Composting

**Table 6:** Barriers to food waste reduction (B).

Quotes from the Interviews	Subtheme	Theme
*“As far as I know, you can only donate food in shopping centres, after you have finished shopping, you can put oil, sugar, flour, etc. in the collection.”*	Lack of information	Awareness and knowledge
*“Also, most of the shops in the leftovers app are just a publicity stunt.”*	Mistrust of information	
*“ The biggest problem is that we buy too much, all at once, like there’s no tomorrow, and then we buy too much in bulk.”*	Stockpiling, hoarding	Behavioral
*“More often than not, I forget to buy exactly what I really need and buy several unnecessary things instead.”*	Lack of consciousness	
*“I try to make a weekly menu, but it doesn’t always work because of the kids.”*	Changing expectations	Logistic challenges
*“Vegetables go bad quickly, so I don’t have time to cook.”*	Lack of time	Economic
*“What goes in the fridge once doesn’t come out, it just goes in the bin, it’s just hard to keep track of what’s left and what’s gone.”*	Lack of effort	
*“Food wastage could be prevented if the programme always went as planned, but because we are young, things can happen spontaneously.”*	Age, motivation	Norms

## Data Availability

The original contributions presented in this study are included in the article. Further inquiries can be directed to the corresponding author.

## Abbreviations

The following abbreviations are used in this manuscript:

TPB	Theory of Planned Behavior
NAM	Norm Activation Model
AR	Ascription of responsibility
AC	Awareness of consequences
PN	Personal norms
DC	Demographic characteristics
FWBI	Food waste behavioral intentions
B	Barriers

## Appendix A. In-Depth Interview Questions

1. How much food do you estimate you throw away in an average week? (e.g., in portions, weight, or number of items)
2. How often do you throw away food items in a typical week? Can you describe the circumstances under which you tend to discard the most food?
3. Are there specific meals (e.g., breakfast, lunch, dinner) or food occasions where you feel more food is wasted?
4. Which types of food do you most often throw away? (e.g., fruits, vegetables, dairy, baked goods, leftovers)
5. Are there particular categories of food that you tend to over-purchase and subsequently waste?
6. Do you notice any difference in food waste for fresh versus packaged or frozen items?
7. Do you notice any patterns in your food waste habits, such as wasting more food at certain times (e.g., weekends, holidays)?
8. Do you believe that food waste is more of an individual or a collective responsibility? Why?
9. How do you perceive your responsibility in reducing food waste?
10. Can you describe situations where you feel more responsible for minimizing food waste (e.g., at home, at work, or in social settings)?
11. What motivates you to take responsibility for reducing food waste (e.g., environmental, financial, or moral concerns)?
12. What do you know about the environmental, social, or economic consequences of food waste?
13. Do you consider the consequences of food waste when making decisions about purchasing or discarding food? Why or why not?
14. Have you encountered any educational programs or resources (e.g., media, schools) that influenced your awareness of food waste? If so, what was the most impactful?
15. How do you think food waste today will affect future generations?
16. How has your upbringing or family environment influenced your attitude toward food waste?
17. Have your thoughts regarding food waste changed over time due to exposure to new information, technology, or education? How? Do you feel guilty or uncomfortable when discarding food? Why or why not?
18. How do you feel when you have to discard food? (guilt or uncomfortable) Why or why not?
19. Do you have any strategies to reduce food waste in your household? Please specify them!
20. Do you usually plan your shopping and cooking? If yes, how?
21. Do you have any strategies to store food properly and prevent spoilage? Please specify them!
22. Have you ever donated leftover food or shared it with others? What motivates or prevents you from doing so?
23. Do you use any technological solutions, such as food rescue apps or community platforms, to minimize waste?
24. What challenges do you face in planning your food purchases or meals to avoid waste?
25. Are there specific types of food that you find more challenging to manage (e.g., perishables, leftovers)?
26. Have you ever encountered difficulties in storing food properly? If so, what were the main challenges?
27. Do you find it difficult to access information or tools to reduce food waste (e.g., donation platforms, composting)?
28. What do you think could help you reduce food waste more effectively (e.g., education, tools, community initiatives)?
29. Are there specific types of support (e.g., government policies, retailer programs) that you think would make food waste reduction easier for households?
30. What role do you think businesses or technology (e.g., apps, innovative packaging) can play in reducing food waste?
31. Reflecting on your habits, what do you think is the most significant driver of your food waste behavior?
32. What advice would you give to others to help them minimize food waste?
33. What do you think needs to change at a societal level to achieve meaningful reductions in food waste?
